# Prevalence and analysis of tobacco use disorder in patients diagnosed with lung cancer

**DOI:** 10.1371/journal.pone.0220127

**Published:** 2019-09-06

**Authors:** Soo-Hyun Paik, Chang Dong Yeo, Jo-Eun Jeong, Ju Sang Kim, Sang Haak Lee, Seung Joon Kim, Dai-Jin Kim

**Affiliations:** 1 Addiction Center, Keyo Hospital, Ojeon-ro, Uiwang-city, Gyeonggi-do, Republic of Korea; 2 Division of Pulmonology, Department of Internal Medicine, The Cancer Research Institute, College of Medicine, The Catholic University of Korea, Banpo-daero, Seocho-gu, Seoul, Republic of Korea; 3 Department of Psychiatry, Seoul St. Mary’s Hospital, College of Medicine, The Catholic University of Korea, Banpo-daero, Seocho-gu, Seoul, Republic of Korea; University of Toronto, CANADA

## Abstract

**Introduction:**

Tobacco use disorder (TUD), previously known as nicotine dependence, was associated with increased risk of lung cancer. However, little is known about the prevalence of TUD and symptom manifestation in smokers with lung cancer.

**Objectives:**

The aim of the present study was to investigate the prevalence of TUD using DSM-5 diagnostic criteria in patients diagnosed with lung cancer and analyze their tobacco use characteristics.

**Methods:**

A total of 200 histologically confirmed lung cancer patients who used tobacco within the prior 12-month period at the time of diagnosis were recruited for this study. Participants were assessed using interviewer-administered questionnaires to determine TUD symptoms and smoking-related behaviors, and self-administered Fagerstrom Test for Nicotine Dependence (FTND) was also administered.

**Results:**

The prevalence of DSM-5 TUD was 92.0% (n = 184). Of a total of 200 subjects, 23 (11.5%), 35 (17.5%), and 126 (63.0%) were classified into mild, moderate, and severe TUD categories, respectively. A total of 19 (81.3%) moderate TUD and 98 (77.8%) severe TUD patients had attempted smoking cessation. Of these subjects, 21 (21.4%) severe TUD and 12 (63.2%) moderate TUD patients tried more than three times. The number of satisfied criteria under DSM-5 TUD was positively correlated with FTND score, cumulative lifetime smoking amount, and daily smoking levels.

**Conclusions:**

Smokers diagnosed with lung cancer showed a high prevalence of DSM-5 TUD. Their heavy and consistent tobacco use suggests reduced motivation to abstain from smoking.

## Introduction

Cigarette smoking is responsible for 70% to 90% of lung cancer [1, 2]. Both smoking duration and amount are positively correlated with increased risk of morbidity and mortality [3–5]. The Liverpool Lung Project (LLP) model has suggested that adjusted odds ratio (OR) is 2.16 in smokers with 1 to 20 years of smoking duration, 4.27 in those with 21 to 40 years of smoking, 12.27 in those with 41 to 60 years of smoking, and 15.25 in those with 60 or more years of smoking compared with never-smokers, indicating a dose-dependent effect of smoking duration [3]. A prospective cohort study conducted in Great Britain has demonstrated an upward trend in the dose-response relationship between cigarettes smoked per day and the incidence of lung cancer [6].

Nonetheless, many patients cannot abandon smoking even after a diagnosis of cancer. Lung cancer patients who are referred to treatment for nicotine dependence have difficulty in maintaining long-term tobacco abstinence despite their high degree of motivation for smoking cessation [7, 8]. Continued tobacco use despite awareness of persistent or recurrent physical or psychological ailments is one of the diagnostic criteria underlying tobacco use disorder (TUD) described in the Diagnostic and Statistical Manual of Mental Disorder, 5^th^ edition (DSM-5) and nicotine dependence of DSM-IV [9, 10]. Thus, tobacco users with a diagnosis of lung cancer may manifest considerably high prevalence of TUD. In the Czech Women’s Lung Cancer case-control study, nicotine dependence was associated with an increased risk of lung cancer [11]. Although many studies have attempted to elucidate the association between TUD and lung cancer, little is known about the prevalence of TUD and symptom manifestation in smokers diagnosed with lung cancer. It seems worthwhile to investigate these issues in light of the recent revisions to the diagnostic criteria based on DSM-IV nicotine dependence to DSM-5 TUD. Therefore, the objectives of this study were to investigate the prevalence of TUD in tobacco users diagnosed with lung cancer under DSM-5 criteria, and determine their tobacco use characteristics. Results of this study might contribute to valuable insights into factors associated with TUD in patients diagnosed with lung cancer and facilitate the development of effective smoking cessation interventions for such patients.

## Materials and methods

### Participants and procedures

We recruited current smokers with histologically confirmed lung cancer presenting at the Departments of Pulmonology of Seoul St. Mary’s Hospital, Incheon St. Mary’s Hospital, St. Paul Hospital, and Uijeongbu St. Mary’s Hospital between January and November of 2017. Current smokers were defined as those with tobacco use during the prior 12-month period at the time of diagnosis according to previous studies [12, 13]. A total of 200 participants were recruited, including 185 males and 15 females.

All participants were assessed via detailed interviewer-administered questionnaires and self-administered Fagerstrom Test for Nicotine dependence (FTND). All interviewers were trained in procedures to standardize data collection and coding techniques, and techniques to minimize inter-interviewer variation. The majority of in-person interviews took 20 to 30 minutes to complete. Data pertaining to histological types and stages of lung cancer were collected from the Catholic Medical Center lung cancer registry.

All study procedures were performed in accordance with the guidelines of the Declaration of Helsinki. This study was approved by the Institutional Review Board of Catholic Medical Center (approval number: XC16QIMI0059). All participants provided written, informed consent.

### Measures

Interviewer-administered questionnaire included demographic characteristics, tobacco use behaviors, and each symptom of DSM-5 TUD and DSM-IV nicotine dependence (ND).

#### Demographic information

Information pertaining to marital and current occupational status was obtained. Marital status was determined as currently living with a spouse (married, cohabited, or remarried) or currently not living with a spouse (never married, divorced, separated, or widowed). Based on current occupational status, participants were deemed as currently employed (either full-time or part-time), unemployed, or other unspecified status.

#### Tobacco use characteristics

A complete smoking history, including smoking onset age, cigarettes smoked per day, cumulative lifetime smoking amount in pack-years, and information related to smoking cessation attempts was obtained. Information regarding smoking cessation attempts included the following questions: 1) the number of attempts (once, twice, or more than three times); 2) the longest duration of smoking cessation, with an answer ranging from < 3 months to ≥ 3 months; 3) exposure to smoking cessation treatment; and 4) any treatment interventions and types of smoking cessation with multiple-alternative answers ranging from counseling, nicotine replacement therapy (NRT), to pharmacotherapy.

#### Defining tobacco use disorder

Since all criteria of DSM-IV ND overlapped with those of DSM-5 TUD, questionnaires included 11 DSM-5 TUD criteria (S1 Table). All interviewers were provided with instructions for comprehensive questions regarding each criterion. Since most patients tended to stop or at least reduce their smoking just before they visited hospital, subjects were asked to respond based on their usual smoking behavior during 12 months prior to the diagnosis of lung cancer. According to the proposed DSM-5 threshold [9], TUD was defined as endorsement of two or more out of 11 DSM-5 TUD criteria. TUD severity was specified as mild (2 to 3 symptoms), moderate (4 to 5 symptoms), or severe (6 or more symptoms). Subjects who satisfied one or less DSM-5 TUD criteria were defined as non-TUD. ND was defined by three or more symptoms of DSM-IV ND criteria according to the threshold [10]. The frequency of each item in DSM-5 TUD was also determined.

#### Fagerstrom test for nicotine dependence (FTND)

FTND, a 6-item self-rated scale with a total score ranging from 0 to 10, was used to assess the severity of ND. According to previous studies [12, 14–16], severe nicotine dependence was defined by a score of 7 or higher. The standardized Korean version of FTND was used in this study. It showed good internal consistency (Cronbach’s alpha = .6913) and validity [16]. A Cronbach’s alpha was .596 in this study.

### Statistical analysis

Descriptive statistics were used to characterize demographics, tobacco use behaviors, and prevalence of tobacco use disorder. Quantitative data are presented as mean ± standard deviation (SD) while qualitative data are presented as absolute number (N) and percentage (%). Comparisons of tobacco use behaviors and frequencies of each item of DSM-5 TUD across non-TUD, mild, moderate, and severe TUD groups were performed using χ^2^ test for nominal variables and Kruskal-Wallis test for continuous variables. Post-hoc analyses were conducted via Bonferroni Correction. Partial correlation analyses using age as a covariate were conducted to explore the relationship between the levels of cumulative lifetime smoking and cigarettes smoked per day, and the number of satisfied criteria of TUD and FTND score. All statistical analyses were performed using SPSS version 24.0 (SPSS Inc., Chicago, IL, USA). Statistical significance was considered at *p* < 0.05.

## Results

### Demographic and tobacco use characteristics and prevalence of tobacco use disorder

Table 1 presents patient demographics. The patients’ age ranged from 35 to 87 years, with a mean age of 65.9 ± 9.6 years. The mean education of the whole sample was 11.0 ± 4.3 years. Of a total of 200 subjects, 172 (86.0%) were currently living with a spouse (married, cohabited, or remarried) and 37 (18.5%) were currently employed. Adenocarcinoma and squamous cell carcinoma were the most frequently observed histological types (n = 80, 40.0%, respectively), followed by small cell carcinoma (n = 29, 14.5%). Two subjects, one with pleomorphic carcinoma and one with adenoid cystic carcinoma, were classified as “others”. Among patients with non-small cell lung cancer (n = 171), 40 (23.4%) subjects were diagnosed with stage I, 17 (9.9%) with stage II, 54 (31.6%) with stage III, and 60 (35.1%) with stage IV disease. Among small cell carcinoma patients (n = 29), 7 (24.1%) were classified as limited stage and 22 (75.9%) were classified as extensive stage.

**Table 1:** Patient demographics.

Age	65.9±9.6
Male (%)	185 (92.5%)
Education year	11.0±4.3
Marital status	
Currently living with a spouse	172 (86.0%)
Currently not living with a spouse	28 (14.0%)
Current occupational status	
Currently having a job	37 (18.5%)
Current not having a job	125 (62.5%)
Others	38 (19.0%)
Histological types	
Adenocarcinoma	80 (40.0%)
Squamous cell carcinoma	80 (40.0%)
Adenosquamous cell carcinoma	1 (0.5%)
Small cell carcinoma	29 (14.5%)
Large cell neuroendocrine carcinoma	2 (1.0%)
Non-small cell carcinoma-NOS	6 (3.0%)
Others	2 (1.0%)
Stages of non-small cell lung cancer (n = 171)	
I	40 (23.4%)
II	17 (9.9%)
III	54 (31.6%)
IV	60 (35.1%)
Stages of small cell lung cancer (n = 29)	
Limited stage	7 (24.1%)
Extensive stage	22 (75.9%)
Smoking onset age (years)	21.9±6.1
Cigarettes per day	21.0±9.1
Cumulative smoking amount (pack-year)	39.7±20.7
FTND score	5.0±2.3
Severe nicotine dependence by FTND (≥7 scores)	55 (27.5%)
DSM-5 Tobacco use disorder	184 (92.0%)
Mild	23 (11.5%)
Moderate	35 (17.5%)
Severe	126 (63.0%)
DSM-IV Nicotine dependence	157 (78.5%)

FTND: Fagerstrom Test for Nicotine Dependence

Subjects started to smoke at an age of 21.9 ± 6.1 years. They smoked 21.0 ± 9.1 cigarettes per day on average. Cumulative lifetime smoking amount was 39.7±20.7 pack-years. The mean FTND score was 5.0 ± 2.3. A total of 55 (27.5%) patients were diagnosed with severe nicotine dependence (≥ 7 on FTND).

The prevalence of DSM-5 tobacco use disorder was 92.0% (n = 184). Of 200 subjects, 23 (11.5%), 35 (17.5%), and 126 (63.0%) were classified as mild, moderate, and severe TUD, respectively. A total of 157 (78.5%) subjects showed DSM-IV nicotine dependence, and 141 (70.5%) manifested nicotine dependence physiologically (withdrawal or tolerance).

A total of 146 (73.0%) subjects had attempted smoking cessation (not shown in the table). These subjects included 63 (31.5%) who attempted once, 41 (20.5%) twice, and 42 (21.0%) more than three times. Eighty-five (42.5%) patients succeeded with smoking cessation within three months while 61 (30.5%) succeeded in ≥ 3 months. Sixty (30.0%) subjects had received smoking cessation treatment: 24 (12.0%) underwent counselling, 9 (4.5%) were exposed to nicotine replacement therapy, 3 (1.5%) received pharmacotherapy, 20 (10.0%) were exposed to a combination of counseling and NRT, 2 (1.0%) underwent counseling combined with pharmacotherapy, and 1 (0.5%) was treated with a combination of counseling, NRT, and pharmacotherapy.

The most frequently observed symptoms of DSM-5 TUD in the whole sample were “continued tobacco use despite knowledge of having a persistent or recurrent physical or psychological problem that is likely to have been caused or exacerbated by tobacco” (n = 165, 82.5%) and “a persistent desire or unsuccessful effort to cut down or control tobacco use” (n = 164, 82.0%) whereas the least frequent symptoms were “great deal of time being spent in activities necessary to obtain or use tobacco” (n = 89, 44.5%) and “giving up or reducing important social, occupational, or recreational activities because of tobacco use” (n = 92, 46.0%).

### Comparison of tobacco use characteristics across non-TUD and TUD severity groups

Table 2 shows tobacco use characteristics across non-TUD, mild, moderate, and severe TUD categories. Cigarettes smoked per day and cumulative lifetime smoking were significantly higher in the severe TUD group, although they were insignificant in post-hoc Bonferroni correction. The mean FTND score was significantly higher in the severe TUD group than in the non-TUD or moderate TUD groups (*p* = 0.001). Attempts at smoking cessation differed significantly across groups. Thirteen (81.3%) non-TUD and 98 (77.8%) severe TUD patients had attempted smoking cessation. However, only 21 (21.4%) severe TUD patients attempted more than three times compared with 12 (63.2%) moderate TUD patients who tried more than three times. Duration of the longest smoking cessation and experience with smoking cessation treatment were similar across groups.

**Table 2:** Comparison of tobacco use characteristics.

	Non-TUD (N = 16)	Mild (N = 23)	Moderate (N = 35)	Severe (N = 126)	*p*-value
Smoking onset age (years old)	20.5±3.2	23.0±6.4	22.1±4.6	21.7±6.6	0.157
Cigarettes per day	19.1±11.8	20.3±7.4	17.9±7.6	22.0±9.3	0.002
Cumulative lifetime smoking amount (pack-year)	30.3±14.3	32.1±12.3	33.57±17.5	43.9±22.4	0.034
FTND score	3.8±2.4	4.6±2.2	3.9±2.2	5.5±2.2	0.001
Attempts of smoking cessation	13 (81.3%)	16 (69.6%)	19 (54.3%)	98 (77.8%)	0.049
Once	5 (38.5%)	6 (37.5%)	5 (26.3%)	47 (48.0%)	0.009
Twice	3 (23.1%)	6 (37.5%)	2 (10.5%)	30 (30.6%)	
More than three times	5 (38.5%)	4 (25.0%)	12 (63.2%)	21 (21.4%)	
Duration of longest smoking cessation (n = 146)					0.092
Less than 3 months	7 (43.8%)	14 (60.9%)	26 (74.3%)	92 (73.0%)	
Over 3 months	9 (56.3%)	9 (39.1%)	9 (25.7%)	34 (27.0%)	
Having smoking cessation treatment	2 (12.5%)	8 (36.4%)	12 (37.5%)	38 (29.8%)	0.269

TUD: Tobacco Use Disorder, FTND: Fagerstrom Test for Nicotine Dependence, NRT: Nicotine Replacement Therapy.

Table 3 shows the frequency of each symptom of the DSM-5 TUD across groups. Since the number of satisfying criteria was used to determine non-, mild, moderate, and severe TUD cases, the frequency was increased with increasing severity. Differences in frequency among groups were statistically significant. In all groups, the two most frequently reported symptoms included “item 6: continued tobacco use despite knowledge of having a persistent or recurrent physical or psychological problem” and “item 2: a persistent desire or unsuccessful effort to cut down or control tobacco use”. Analysis of the physiological dependence symptoms suggested that one (6.3%) patient with non-TUD, 4 (17.4%) with mild TUD, 8 (22.2%) with moderate TUD, and 99 (78.6%) with severe TUD reported tolerance while 3 (13.0%) in the mild TUD, 20 (57.1%) in the moderate TUD, and 93 (73.8%) in the severe TUD groups reported withdrawal.

**Table 3:** Comparative frequencies of each symptom of DSM-5 tobacco use disorder.

Symptom criteria	Non-TUD (N = 16)	Mild (N = 23)	Moderate (N = 35)	Severe (N = 126)	*p*-value
Often taken in larger amounts or over a longer period than was intended	0 (0.0%)	0 (0.0%)	12 (34.3%)	85 (67.5%)	0.001
A persistent desire or unsuccessful efforts to cut down or control tobacco use.	4 (25.0%)	11 (47.8%)	31 (88.6%)	118 (93.7%)	0.001
A great deal of time being spent in activities necessary to obtain or use tobacco	0 (0.0%)	2 (8.7%)	8 (22.9%)	79 (62.7%)	0.001
Craving or strong desire or urge to uses tobacco	1 (6.3%)	10 (43.5%)	18 (51.4%)	108 (85.7%)	0.001
Recurrent tobacco use resulting in a failure to fulfill major role obligations at work, school, or home	0 (0.0%)	4 (17.4%)	8 (22.9%)	87 (69.0%)	0.001
Continued tobacco use despite having persistent or recurrent social or interpersonal problems caused or exacerbated by the effects of tobacco.	0 (0.0%)	4 (17.4%)	16 (45.7%)	113 (89.7%)	0.001
Giving up or reducing important social, occupational, or recreational activities because of tobacco use.	0 (0.0%)	5 (21.7%)	8 (22.9%)	79 (62.7%)	0.001
Recurrent tobacco use in situations in which it is physically hazardous	1 (6.3%)	2 (8.7%)	4 (11.4%)	91 (72.2%)	0.001
Continued tobacco use despite knowledge of having a persistent or recurrent physical or psychological problem that is likely to have been caused or exacerbated by tobacco.	3 (18.8%)	13 (56.5%)	27 (77.1%)	122 (96.8%)	0.001
Tolerance	1 (6.3%)	4 (17.4%)	8 (22.2%)	99 (78.6%)	0.001
Withdrawal	0 (0.0%)	3 (13.0%)	20 (57.1%)	93 (73.8%)	0.001

When the frequency of the FTND items in the whole sample was evaluated for the use of the first morning cigarette of the day (item 1: time to smoke first cigarette; item 3: hating most to give up the first cigarette in the morning; and item 5: smoke more frequently during the first hour after waking than during the rest of the day), 85 (42.5%) patients smoked the first cigarette within 5 minutes, 59 (29.5%) in 6 to 30 minutes, 29 (14.5%) in 31 to 60 minutes, and 27 (13.5%) in more than 60 minutes after waking up. However, 113 (56.5%) answered they hated the most to give up the first cigarette in the morning and 65 (32.5%) smoked more frequently during the first hour after waking than during the rest of the day (S2 Table). There were no group differences in these three items. Frequencies of the other three items (item 2: difficulty refraining from smoking in places where it is forbidden; item 4: amount of cigarettes per day; and item 6: smoke if they are so ill that they are in bed most of the day) were the highest in the severe TUD group and the lowest in the non-TUD group.

### Correlation between the severity of tobacco use disorder and smoking amount

Table 4 shows results of partial correlation analyses between smoking amount and the severity of TUD using age as a covariate. The number of satisfied criteria of DSM-5 TUD was positively correlated with cumulative lifetime smoking amount (r = 0.351, *p* = 0.001), cigarettes per day (r = 0.152, *p* = 0.032), and FTND score (r = 0.349, *p* = 0.001).

**Table 4:** Pearson correlation coefficient between smoking amount and severity of tobacco use disorder (age-adjusted).

	Severity of tobacco use disorder			
	1	2	3	4
Cumulative lifetime smoking amount	1			
Average cigarettes per day	0.705**	1		
DSM-5^¶^	0.351**	0.152*	1	
FTND score	0.424**	0.420**	0.349**	1

FTND: Fagerstrom Test for Nicotine Dependence.

^¶^ Number of satisfied criteria of DSM-5 tobacco use disorder.

* p<0.05

** p <0.005.

## Discussion

This study explored the prevalence of DSM-5 TUD in tobacco users diagnosed with lung cancer and comprehensively analyzed the tobacco use characteristics. We found that the prevalence of TUD was 92.0% and 63.0% in patients with severe TUD. Tobacco use characteristics differed across the severity groups. In addition, heavy and consistent tobacco use was associated with great difficulty in quitting and abstinence from smoking in patients with lung cancers.

*Mikami* et al. [12] have reported that the prevalence of ND according to DSM, 3^rd^ edition, revised (DSM-III-R) was 67.0% among 151 lung and head and neck cancer patients with a 1-year smoking addiction [12]. Although direct comparison was impossible due to the differences in sample recruitment, measurements, and definition of current smoking status, the prevalence of TUD based on DSM-5 criteria in smokers with lung cancer was very high (92.0%) in our study.

The prevalence of DSM-IV nicotine dependence was 78.5% in this study, which was lower than that of DSM-5 TUD (92.0%). Overdiagnosis has been a major concern of DSM-5 TUD since the diagnostic threshold declined from 3 out of 7 criteria to 2 out of 11 [13, 17]. However, these changes were intended to address the concerns of individuals who showed harmful smoking patterns not yet diagnosed as dependent based on DSM-IV, and to improve sensitivity, specificity, and predictive value of poor results under the DSM-IV ND criteria [18–20]. Diagnostic cases of TUD under DSM-IV criteria were detected using the revised DSM-5 criteria in adolescent samples, as well. Taken together, the gap between the prevalence of DSM-5 TUD and DSM-IV ND reflects veiled tobacco use in tobacco users diagnosed with lung cancer.

The prevalence of TUD in smokers with lung cancer was 92.0% in this study. A systematic review of the prevalence of DSM/ICD-defined nicotine dependence in the general population demonstrated that a third of ever-smokers and 50% of current smokers either have been or are currently dependent on nicotine irrespective of age, country, or sex and that 50% of current smokers do not meet DSM/ICD dependence criteria [21]. Though direct comparison is impossible, the prevalence and characteristics of TUD in lung cancer patients might differ from that of the general population. It is plausible that the general population includes a large number of non-daily smokers who were less likely to become dependent [22]. *Okuyemi* et al. [23] suggested that light smokers may need varying levels of nicotine dependence rating scale. By contrast, previous studies showed that many patients diagnosed with smoking-related cancer have a history of heavy and consistent smoking [4, 5]. In this study, subjects reported smoking more than 1 pack per day on average and their cumulative smoking level was almost 40 pack-years. Large amount and long duration of smoking, which are major risk factors and consequences of tobacco use disorder, might have contributed to the development of lung cancer. The general population may deny their dependent status while cancer patients may confess or at least recognize their dependency on tobacco after their diagnoses. Taken together, tobacco users diagnosed with lung cancer exhibit different patterns of TUD prevalence compared with the general population possibly due to their heavy and consistent tobacco use and awareness of dependency.

In this study, although 73.0% of subjects attempted smoking cessation at least once and 30.0% received interventions, all subjects failed to quit completely in the previous 12 months. Considering that a substantial number of smokers with cancer could not quit smoking after cancer diagnosis and treatment despite their interest in smoking cessation interventions and multiple trials [7, 24, 25], it can be inferred that current smokers with lung cancer suffer from severe TUD and its consequences compared with cancer-free subjects who were current smokers. In addition, the number and frequency of attempts were different across groups. Although the difference in the longest duration of smoking cessation did not statistically differ, these findings suggested that subjects with severe TUD might be less motivated to fail to abstain than mild or moderate cases of TUD. It is well known that chronic smoking induces neuroadaptation, which interferes with behavioral changes [26]. Accordingly, it is essential to tailor TUD intervention for current smokers with lung cancer [27, 28]. For example, according to Chun, the combination of counseling and pharmacotherapy in patients with chronic obstructive pulmonary disease (COPD) increased the quitting rate more than twice than behavioral intervention alone. We suggest that TUD patients with lung cancer may need intensive and individualized strategies, including motivation enhancement and maintenance, and strategies for the management of craving symptoms [28, 29].

A loss of control over tobacco use and a persistent desire or unsuccessful effort to cut down or control tobacco use have been uniformly high in patients with ND as well as non-dependent subjects in the general population [30, 31]. This finding implies that loss of control may be the most salient symptom of tobacco use irrespective of physical condition or degree of dependency and may not show discrimination between non-TUD and TUD. Conversely, the frequency of the item “continued use despite knowledge” was high in this study (82.5%). In contrast, the frequency observed in the general population with ND ranged from 40% to 60% [30–32]. While these results need cautious interpretation, current smokers with lung cancers might have difficulty in cutting down smoking despite their physical and psychological problems or knowledge of adverse health consequences. This finding is a cardinal feature of TUD in smokers with lung cancers and a critical decision point for intensive and tailored smoking cessation intervention for lung cancer patients. More than 70% of severe TUD showed physiological dependence symptoms, either tolerance or withdrawal, whereas the frequency of these symptoms was not as high in other groups. This finding was in consistent with previous studies demonstrating that physiological dependence was less frequent in non-dependent smokers [31].

The distribution of TUD severity measured via DSM-5 and FTND varied significantly in this study. While severe TUD examined under DSM-5 criteria was 63.0%, severe ND measured using FTND was 27.5%. A possible explanation for the discrepancy observed in this study is tobacco users with lung cancers may present more psychological symptoms or associated functional impairment than physiological dependence. FTND consists of three items measuring physiological dependence (items 1, 4, and 5) and three behavioral manifestations (items 2, 3, and 6) whereas DSM-5 TUD criteria comprise additional items to assess psychological symptoms and functional impairment. *Moolchan* et al. [15] have suggested that FTND may suggest a strong physical dependence while DSM may tap other domains such as awareness of dependence, behaviors, and psychiatric symptoms, suggesting that FTND and DSM criteria can be used to assess different aspects of tobacco dependence [33]. Further, the recruited subjects might be unaware of their physiological dependence due to their reactive anxiety or depression following the diagnosis of lung cancer. Tobacco withdrawal symptoms share common manifestations with these psychiatric conditions. Meanwhile, two previous studies that investigated patients with smoking-related cancer showed a higher mean FTND score (7.5±1.9, and 5.8±2.3) and prevalence of nicotine dependence (73.2% with FTND 7–10 scores, and 66.2% with FTND 6–10 scores) [14, 34] compared with our study (mean FTND score: 5.0±2.3; 27.5% with FTND 7–10 scores). While both studies included patients with cancer undergoing regular outpatient follow-up, subjects in this study visited tertiary hospitals to histologically confirm lung cancer. Such patients might be apprehensive of the worst outcome, and might have at least stopped or reduced smoking for several days that reduced the awareness of their physiological dependence.

We found that the number of satisfied criteria under DSM-5 TUD was positively correlated with smoking levels, both lifetime cumulative (pack-year) and daily cigarettes, and FTND scores. This finding was consistent with previous studies reporting a correlation between FTND score and smoking amount [12, 17], demonstrating the utility of the severity specifier proposed in DSM-5.

This study has several limitations. First, smoking amounts were determined only based on self-reported questionnaires, which may be underestimated or overestimated. Usually, underestimation is a major issue since dependent individuals deny or minimize their problem. Nonetheless, 92.0% of subjects showed TUD, which suggested that most, if not all, of the current smoking-related cases of lung cancer may show TUD with varying severity. Second, the distribution of non-TUD and the TUD groups varied significantly in severity. However, it suggests that current smokers with lung cancers suffer from severe TUD. Third, since our study did not involve currently smoking healthy controls or non-smoking lung cancer patients, a direct comparison with the prevalence in general population is difficult. Further, the sample may be biased toward the most dependent nicotine users since they failed to quit smoking in the previous 12 months despite their attempts to quit (73.0% tried quitting), strongly suggesting that the prevalence of TUD, especially with severity, is particularly high in tobacco users with lung cancer. Further studies should include previous smokers who quitted smoking more than in the previous 12 months to evaluate this hypothesis. Fourth, this was a cross-sectional study. A longitudinal follow-up to determine changes in diagnosis or severity is needed in the future. Lastly, the time point for the cancer diagnosis is unclear.

Despite the limitations, the strengths of the study outweigh the limitations for the following reasons: 1) the sample was homogenous in that they all had histologically confirmed lung cancer; 2) this study investigated each symptom of TUD and each item of FTND as well as tobacco use characteristics comprehensively; and 3) to the best of our knowledge, this is the first study to investigate the prevalence of DSM-5 TUD and DSM-IV ND simultaneously in a lung cancer population. These results may shed light on the factors underlying the severity of TUD in patients diagnosed with lung cancer, for intervention with effective smoking cessation strategies.

## Conclusion

We demonstrated that tobacco users diagnosed with lung cancer had a high prevalence of TUD based on DSM-5 criteria. Heavy and consistent tobacco use in patients with lung cancers might dampen their motivation to quit and reduce their adherence with tobacco cessation interventions. These results have an important clinical implication for health care providers in that lung cancer patients might carry severe tobacco use problems, warranting intensive and individualized therapeutic strategies for smoking cessation, especially for those with psychological symptoms.

## Supporting information

S1 Table Lifetime DSM-5 TUD and DSM-IV criteria. (DOCX)

S2 Table Comparisons of frequencies of each item of Fagerstrom test for nicotine ependence. (DOCX)

S1 Data (XLSX)
