# Supplementary material for: Prevalence and analysis of tobacco use disorder in patients diagnosed with lung cancer
Source: PLoS One. 2019 Sep 6;14(9):e0220127. doi: 10.1371/journal.pone.0220127 (PMC6730883; doi:10.1371/journal.pone.0220127)
Supplement: S1 Table — (DOCX) [file pone.0220127.s001.docx]

**S1 Table. Lifetime DSM-5 TUD and DSM-IV criteria**

|  | DSM-5 TUD | DSM-IV ND |
| --- | --- | --- |
| 1. Often taken in larger amounts or over a longer period than was intended | V | V |
| 2. A persistent desire or unsuccessful efforts to cut down or control tobacco use. | V | V |
| 3. A great deal of time being spent in activities necessary to obtain or use tobacco | V | V |
| 4. Craving or strong desire or urge to uses tobacco | V |  |
| 5. Recurrent tobacco use resulting in a failure to fulfill major role obligations at work, school, or home | V |  |
| 6. Continued tobacco use despite having persistent or recurrent social or interpersonal problems caused or exacerbated by the effects of tobacco. | V | V |
| 7. Giving up or reducing important social, occupational, or recreational activities because of tobacco use. | V | V |
| 8. Recurrent tobacco use in situations in which it is physically hazardous | V |  |
| 9. Continued tobacco use despite knowledge of having a persistent or recurrent physical or psychological problem that is likely to have been caused or exacerbated by tobacco. | V |  |
| 10. Tolerance | V | V |
| 11. Withdrawal | V | V |
| Diagnostic threshold | ≥ 2 items | ≥ 3 items |
