# Supplementary material for: Prevalence and analysis of tobacco use disorder in patients diagnosed with lung cancer
Source: PLoS One. 2019 Sep 6;14(9):e0220127. doi: 10.1371/journal.pone.0220127 (PMC6730883; doi:10.1371/journal.pone.0220127)
Supplement: S2 Table — (DOCX) [file pone.0220127.s002.docx]

**S2 Table. Comparisons of frequencies of each item of Fagerstrom Test for Nicotine Dependence**

|  | Non-TUD  (N=16) | Mild  (N=23) | Moderate (N=35) | Severe (N=126) | *p*-value |
| --- | --- | --- | --- | --- | --- |
| 1. Time to smoke first cigarette |  |  |  |  |  |
| After 60 minutes (score=0) | 2 (12.5%) | 3 (13.0%) | 7 (20.0%) | 15 (11.9%) | .591 |
| 31 to 60 minutes (score=1) | 3 (18.8%) | 4 (17.4%) | 7 (20.0%) | 15 (11.9%) |  |
| 6 to 30 minutes (score=2) | 7 (43.8%) | 5 (21.7%) | 10 (28.6%) | 37 (29.4%) |  |
| Within 5 minutes (score=3) | 4 (25.0%) | 11 (47.8%) | 11 (31.4%) | 59 (46.8%) |  |
| 2. Difficulty in refraining from smoking in place where it is forbidden. (score=1) | 5 (31.3%) | 12 (52.2%) | 9 (25.7%) | 68 (54.0%) | .014 |
| 3. Hating most to give up the first cigarette in the morning (score=1). | 6 (37.5%) | 12 (52.2%) | 17 (48.6%) | 78 (61.9%) | .178 |
| 4. Amount of cigarettes per day |  |  |  |  |  |
| 10 or less (score=0) | 5 (31.3%) | 7 (30.4%) | 12 (34.3%) | 28 (22.2%) | .008 |
| 11 to 20 (score=1) | 10 (62.5%) | 13 (56.5%) | 21 (60.0%) | 56 (44.4%) |  |
| 21 to 30 (score=2) | 1 (6.3%) | 3 (13.0%) | 2 (5.7%) | 32 (26.2%) |  |
| 31 or more (score=3) | 0 (0.0%) | 0 (0.0%) | 0 (0.0%) | 9 (7.1%) |  |
| 5. Smoke more frequently during the first hour after waking than during the rest of the day. (score=1) | 4 (25.0%) | 8 (34.8%) | 8 (22.9%) | 45 (35.7%) | .468 |
| 6. Smoke if you are so ill that you are in bed most of the day. (score=1) | 4 (25.0%) | 7 (30.4%) | 16 (45.7%) | 83 (65.9%) | .001 |
